# ABC Transporter Protein-mediated Multidrug Resistance in Breast Cancer and Development of Targeting Strategies

**DOI:** 10.2174/0115748928394153250731033624

**Published:** 2025-08-12

**Authors:** Xiaoyun Yu, Qihang Shang, Mengrui Yang, Jia Wang, Wenfeng Zhang, Xue Wang, Yan Yao, Jing Zhuang, Changgang Sun

**Affiliations:** 1 College of Traditional Chinese Medicine, Shandong Second Medical University, Weifang, 261053, China;; 2 Department of Oncology, Weifang Traditional Chinese Hospital, Weifang, 261000, China;; 3 College of First Clinical Medicine, Shandong University of Chinese Medicine, Jinan, 250014, China;; 4 Antitumor Omics Laboratory of Traditional Chinese Medicine, Weifang Traditional Chinese Hospital, Weifang, 261000, China;; 5 College of Traditional Chinese Medicine, Shandong University of Chinese Medicine, Jinan, 250014, China

**Keywords:** Breast cancer, ABC transporter protein, multidrug resistance, cell membrane lipid, hypoxia, natural products

## Abstract

Drug resistance (MDR) poses a significant challenge in breast cancer (BrCa) treatment, resulting in reduced efficacy and increased tumor recurrence. Resolving MDR in BrCa is necessary for improving the clinical efficacy of antitumor therapy. However, the molecular mechanisms underlying MDR are complex and involve various biological processes, including ABC drug transporter-mediated drug efflux, abnormal drug metabolism, and the development of the tumor microenvironment. The abnormal expression of ABC transporter proteins constitutes a critical mechanism driving MDR in BrCa. Factors like breast cancer stem cells (BCSCs), epigenetic changes, cell membrane lipids, and microenvironmental components affect the expression and function of ABC transporter proteins in BrCa. Here, we focus on the roles of P-glycoprotein (P-gp), multidrug resistance protein 1 (MRP1), and breast cancer resistance protein (BCRP) in relation to MDR. Additionally, we review the factors affecting the expression and function of these transporters, emphasizing strategies such as gene modification, drug development, and modulation of cell membrane lipids.

## 1 INTRODUCTION

Despite advancements in the treatment of breast cancer (BrCa), multidrug resistance (MDR) remains a significant obstacle to effective treatment for patients with BrCa. In the commonest and most efficient prognostic subtype of BrCa, ER+ BrCa, the selective estrogen receptor modulator (SERM) tamoxifen is the current standard of care and significantly improves overall survival. However, almost 30% of patients who receive tamoxifen develop resistance [1, 2]. Compared with HER2+ and ER+ subtypes, triple-negative breast cancer (TNBC) lacks specific biomarkers and is highly susceptible to chemotherapy resistance. As BrCa cells adapt to therapeutic agents during treatment, the development of MDR in these cells under drug selection pressure-characterized by simultaneous resistance to multiple drugs with varying structures and mechanisms of action-poses a significant challenge in the management of BrCa and is closely associated with elevated mortality rates [3]. Therefore, it is crucial to investigate the underlying mechanisms contributing to MDR in BrCa cells and identify effective strategies aimed at enhancing therapeutic efficacy while improving patient prognosis.

The intricate mechanism of MDR in BrCa involves multiple biological processes, including the dysregulated expression of the transmembrane transporter family, impaired apoptosis in BrCa cells, deletion or abnormal expression of cell-surface hormone receptors, and metabolic clearance of drugs that results in diminished plasma drug exposure. Additionally, factors within the tumor microenvironment-both cellular and secreted-along with metabolic reprogramming, also contribute to this complex phenomenon. Among these factors, the aberrant expression of ATP-binding cassette (ABC) transporter proteins, which are responsible for drug efflux, represents one of the most significant mechanisms contributing to the development of multidrug resistance (MDR), which primarily constitutes the following: 1) drug efflux is mediated by ABC transporter proteins on cell membranes; 2) hypoxic state induces the expression of hypoxia-inducible factor 1 (HIF-1); the HIF-1 transcription factor binds to certain encoding ABC transporter proteins on the hypoxia-responsive elements on genes and activates the expression of multiple drug efflux transporter proteins; 3) epigenetic reprogramming upregulates the expression of ABC transporter proteins; 4) Exosomes convey chemotherapy resistance by delivering P-glycoprotein(P-gp) and miRNA [4]; 5) epithelial-mesenchymal transition (EMT) affects the expression of ABC transporter proteins [5]; and 6) the surface of self-renewal-competent breast cancer stem cells (BCSCs) is highly expressive of ABC transporter proteins [6].

In BrCa cells with an MDR phenotype, overexpressed ABC transporter proteins not only operate independently in drug efflux, but also serve as a "bridge" to other factors that contribute to the development of MDR. During the processes of epigenetic reprogramming, EMT, lipid metabolism reprogramming, and various other mechanisms driving MDR progression, the expression and function of ABC transporters are influenced by these aforementioned pathways, leading to a decrease in intracellular drug concentration. Therefore, elucidating the mechanisms through which ABC transporter proteins facilitate MDR development in BrCa cells is crucial for overcoming MDR and enhancing the survival rates of patients with BrCa. In this review, we provide an update on the pivotal role of ABC transporter proteins in mediating MDR within BrCa cells. We emphasize emerging intervention strategies that focus on molecular expression regulation, modulation of cell membrane lipids, and alteration of the BrCa microenvironment. These approaches aim to target ABC transporter proteins with the goal of reversing MDR in BrCa cells or restoring their sensitivity to therapeutic agents.

### 1.1 Literature Search Strategy

This study employed a systematic retrieval methodology. Initially, comprehensive searches were conducted in databases such as PubMed, Web of Science, and the China National Intellectual Property Administration patent database. Additionally, the back issues of major journals in relevant fields were manually supplemented to ensure completeness. The search time frame was restricted to the past five years, while classical theoretical and foundational studies published prior to this period were selectively included. A total of 15 retrieval expressions were constructed based on Boolean operators (*e.g*., ("ABC transporter*" [TIAB] OR "P-glycoprotein" [TIAB] OR "MDR1" [TIAB] OR "MRP1" [TIAB] OR "BCRP" [TIAB]) AND ("multidrug resistan*" [TIAB] OR "MDR" [TIAB]) AND ("breast cancer" [TIAB] OR "BrCa" [TIAB])), with subject terms matched against free words in the title and abstract.

The literature screening process involved the following steps: (1) After merging and removing duplicates using EndNote, three researchers independently screened titles/abstracts based on inclusion criteria: "ABC transporter" AND ("breast cancer" OR "MDR") AND ("inhibitor" OR "targeting strategy"); Exclude keywords: "non-cancer" OR "single-cell analysis"; (2) Articles that did not involve mechanistic studies, lacked defined models or focused on non-breast cancer research, or failed to provide ABC transporter data or functional validation were excluded; (3) Disputed documents were resolved through arbitration by a fourth researcher. Thematic analysis was subsequently performed to elucidate the drug resistance mechanisms of ABC transporters, regulatory factors such as epigenetics and the tumor microenvironment, as well as the clinical translation potential of targeted strategies including nanocarriers and gene editing.

## 2 ROLE OF ABC TRANSPORTER PROTEINS IN MDR OF BRCA

As one of the largest families of transporter proteins in living organisms, ABC transporter proteins are classified into seven subfamilies (A to G) based on gene structure, domain organization, and sequence homology. These proteins are widely distributed in cell membranes and function primarily as "exporters" [7]. Different ABC transporter proteins exhibit overlapping substrate specificities and are highly heterogeneous [8]. In this study, we focused on three ABC transporter proteins: P-gp (MDR1/ABCB1), Multidrug resistance protein 1 (MRP1/ABCC1), and Breast cancer resistance protein (BCRP/ ABCG2), which are overexpressed in BrCa and closely related to MDR [8, 9].

### 2.1 P-gp

**Fig. (1):**
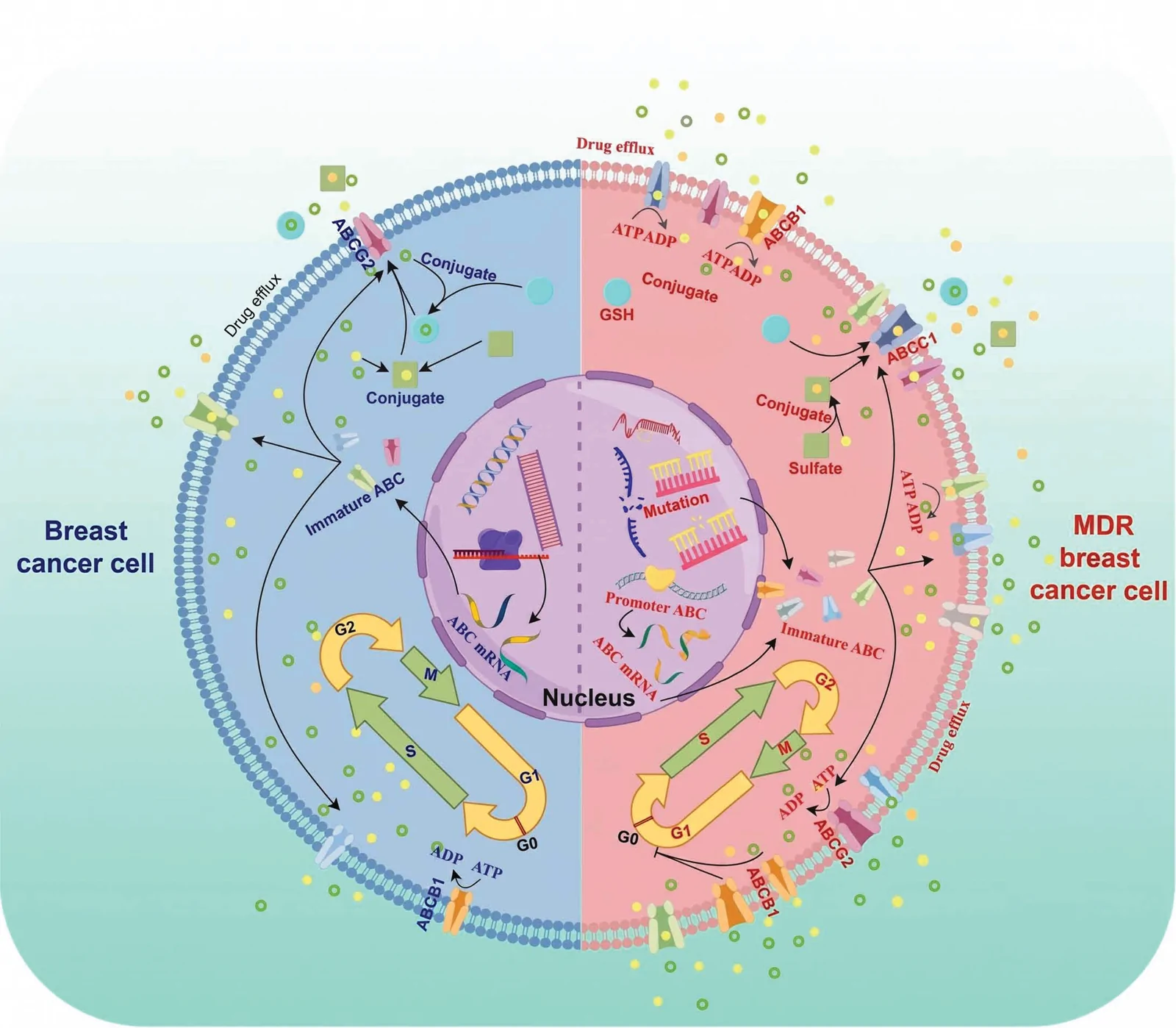
Several mechanisms of ABC transporter mediating MDR in BrCa. (1) Alterations in the genes and transcription factors that encode ABC transporters result in the overexpression of these transporters, leading to an enhanced efflux of drugs. Overexpression of the P-gp can cause abnormal cell cycle of BrCa and make cells resistant to ICIs. (2) MRP1 transporter can combine with GSH to transport organic anion substrate to the extra-cellular. (3) BCRP protein excreted drugs through an extrusion mechanism.

Decreased intracellular drug accumulation is attributed to multiple overlapping substrate-binding sites within the P-gp substrate-binding pocket, as well as the overexpression of P-gp itself. Genetic rearrangements and mutations in the *ABCB1* promoter, along with altered expression and activity of transcription factors, are significant contributors to P-gp overexpression in multidrug-resistant tumor cells [10, 11] (Fig. **1**). Christie *et al*. performed sequencing on recurrent or end-stage samples from 33 BrCa patients who had previously undergone chemotherapy. They detected the SLC25A40-ABCB1 fusion in samples obtained from 9 patients and identified multiple transcriptional fusion partners involving *ABCB1* by placing these samples under a strong promoter while preserving their open reading frames (ORFs). Most currently approved PARP inhibitors (PARPi) are substrates of P-gp. The transcriptional fusion of P-gp may contribute to treatment failure with PARPi and the development of MDR in BrCa [12].

Evidence indicates that when chemotherapeutic drugs enter BrCa cells, the cells initiate a protective mechanism, and the mRNA and protein expression levels of *ABCB1* are elevated. The upregulation of P-gp expression and activity can lead to increased drug efflux and increased efflux efficiency. This phenomenon may result in a decreased effective concentration of drugs within BrCa cells, ultimately contributing to the development of MDR. Boichuk and Galembikova *et al*. showed that heightened P-gp expression in the paclitaxel-resistant (TxR) HCC1806 TNBC cell line significantly contributes to the augmented efflux of paclitaxel and other chemotherapeutic agents [13]. Compared to the parental HCC1806 cell line, HCC1806-TxR cells exhibited a higher IC50 value for paclitaxel (approximately 17-fold) and showed cross-resistance to other types of chemotherapeutic agents, such as vincristine, etoposide, and doxorubicin topoisomerase type II inhibitors. Furthermore, P-gp may facilitate MDR by modifying the cell cycle of BrCa cells (Fig. **1**). Zhang *et al*. showed that P-gp overexpression caused epirubicin-resistant MDA-MB-231 cells to undergo a different cell cycle from their parental MDA-MB-231 cells, and this change may alter the expression and function of the checkpoint proteins, which may facilitate the rapid development of MDR to immune checkpoint inhibitors (ICIs) in patients with TNBC [14].

### 2.2 MRP1

MRP1 overexpression is a contributing factor to the acquisition of a MDR phenotype in metastatic BrCa. In a retrospective study of patients with BrCa, disease progression was observed in 14 of 17 (82.3%) patients whose cell membrane staining showed high MRP1 expression during polychemotherapy (PCT), with a malignant phenotype corresponding to primary MDR status, and 80.0% of patients in this group did not respond to neoadjuvant chemotherapy (NAC) [15].

MRP1 can pump antitumor drugs, such as anthracyclines (doxorubicin, epirubicin, and mitoxantrone), taxanes (docetaxel and paclitaxel), and antifolates (methotrexate) from TNBC cells in an ATP-dependent manner *via* conformational changes in the drug-binding pocket upon binding to substrates. Consequently, the concentration of the effective drug in the cell is reduced, resulting in the attenuation of the drug's antitumor effect and helping the TNBC cells acquire MDR. After analyzing changes in the expression of PTEN, p53, Bcl2, MRP1, and other proteins before and after anthracycline/paclitaxel NAC in 148 patients with TNBC, Guestini *et al*. found that the drugs widely used in the TNBC-NAC regimen are substrates for MRP1, which transports the drugs to the extracellular compartment and drives the progression of drug-resistant TNBC after NAC [16]. Additionally, Loignon *et al*. demonstrated that elevated expression levels of MRP1 in MCF-7 BrCa cells enhanced their capacity to withstand doxorubicin and paclitaxel treatment [17]. Moreover, for certain substrates, MRP1 needs to cooperate with glutathione (GSH) to play its role as a "porter" and various organic anionic substrates of MRP1 need to be conjugated with GSH, glucuronide, or sulfate to be transported to the extracellular compartment (Fig. **1**) [18].

### 2.3 BCRP

In contrast to the two members of the full transporter protein family above, BCRP, a hemi-transporter protein, can squeeze around the substrate, thereby exocytosing the drug (Fig. **1**) [9]. Notably, BCRP serves as an important stem cell marker that is closely associated with drug resistance in BCSC [19]. Compared to the parental MCF-7 cell line, mitoxantrone-resistant MCF-7/MitoR BrCa cells exhibiting overexpression of the *ABCG2* gene product demonstrated a significant reduction in intracellular accumulation of Hoechst33342 dye within tumor cells. Additionally, there was an increase in the population of side population (SP) cells characterized by acquired MDR phenotypes [20]. SP cells have stem cell properties and highly express drug transporter proteins such as BCRP on their surface, which not only expel Hoechst33342 dye out of the cell, but also expel cytotoxic drugs out of the cell and enhances the tolerance of tumor cells to drugs [18]. In addition, using siRNA knockdown and chemosensitivity assays, Mukherjee *et al*. found that the SOX2-ABCG2-TWIST1 axis plays a key role in regulating the chemoresistance and tumorigenicity of TNBC stem cells [21]. SOX2 is recognized as a key marker for stem cells; its silencing resulted in a significant downregulation of BCRP expression and markedly diminished self-renewal capabilities among BCSCs [21]. Moreover, overexpression of TWIST1 was found to contribute to reduced sensitivity of BCSCs towards chemotherapeutic agents such as vincristine, and paclitaxel.

## 3 FACTORS INFLUENCING ABC TRANSPORTER PROTEINS IN THE DEVELOPMENT OF MDR IN BRCA

ABC transporter protein-mediated MDR in BrCa cells is not exclusively the result of structural or functional alterations. Factors such as the high expression of BCSCs, epigenetic modifications, membrane lipid composition, EMT, and various characteristics and components of the microenvironment can directly influence ABC transporter proteins, leading to a cascade of reactions.

### 3.1 Cancer Stem Cells (CSCs)

**Fig. (2):**
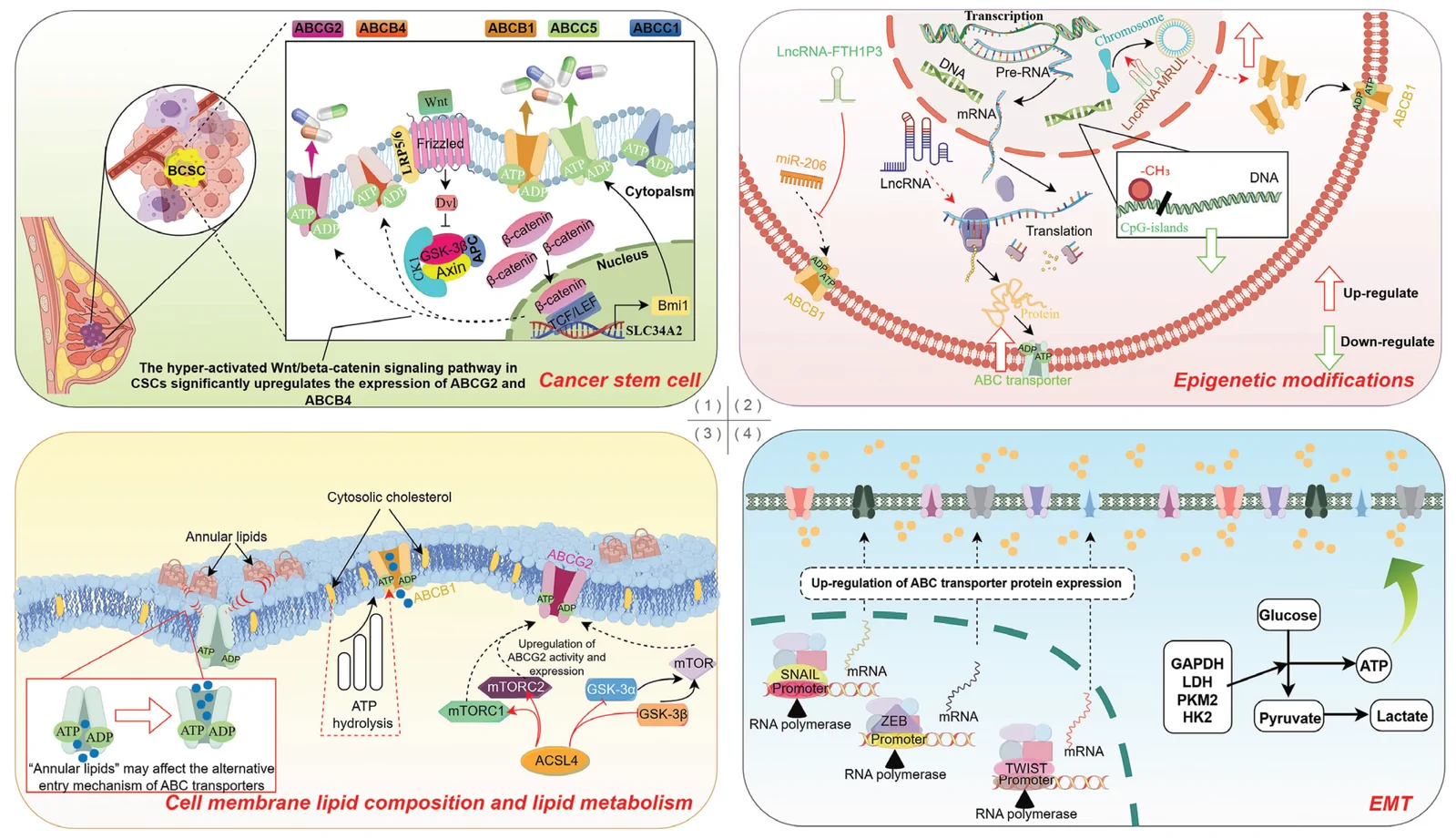
Several factors affect the expression and function of ABC transporter during the occurrence of BrCa MDR. (1) BCSCs exhibit high expression levels of ABC transporters, which contribute to the tolerance of BrCa tissue to drug toxicity. (2) Abnormal expression of non-coding RNAs can upregulate ABC transporters, and alterations in the methylation patterns of transporter promoters influence the responsiveness of BrCa cells to therapeutic agents. (3) Membrane lipid components interact with transporters to enhance drug efflux. Cholesterol accelerates the ATP hydrolysis of P-gp, while the fatty acid metabolism enzyme ACSL4 promotes the expression of BCRP. (4) Certain EMT markers or transcription factors are known to induce the expression of genes that encode ABC transporters. Additionally, glycolysis provides a steady supply of ATP for ABC transporters to work.

BCSCs can mediate BrCa MDR through the overexpression of ABC transporter proteins, particularly in TNBC, where a higher percentage of BCSCs is observed (Fig. **2**). In a tissue biopsy of TNBC following conventional chemotherapy, Creighton *et al*. reported an upregulation of stem cell-related gene expression and a significant increase in the proportion of BCSCs [22]. The cell surface of CSCs highly expresses transporter proteins, such as P-gp, MRP1, BCRP, and ABCB5, particularly BCRP, a phenotypic marker of CSCs [23, 24]. Research has demonstrated that hyperactivation of the Wnt/beta-catenin signaling pathway in CSCs significantly enhances the expression levels of both BCRP and ABCB4 [25]. In addition, another study reported in detail the specific mechanism by which the solute carrier family *SLC34A2* gene induces chemoresistance in BCSCs through the SLC34A2-Bmi1-ABCC5 signaling axis. SLC34A2 regulates the stem cell-associated transcription factor (Bmi1) through the Wnt/beta-catenin pathway [26], whereas ABCC5 serves as a direct Bmi1 transcriptional target, the expression of which was increased under the regulation of upstream molecules, led to more drugs being transported out of BCSCs, causing BCSCs to become resistant to the anthracycline chemotherapeutic drug doxorubicin [27]. Since BCSCs can help BrCa to acquire MDR by high expression of transporter proteins such as BCRP and ABCB4 on the cell membrane, the inhibition of BCSCs can prevent the development of MDR and BrCa recurrence to a certain extent.

### 3.2 Epigenetic Modifications

Aberrant expression of non-coding RNA (ncRNA) can lead to the upregulation of ABC transporter protein levels, and long-chain non-coding RNAs (lncRNAs) inducing MDR in BrCa cells through the upregulation of P-gp (Fig. **2**) [28]. Wang *et al*. discovered that the expression of lncRNA ferritin heavy chain 1 pseudogene 3 (FTH1P3) was significantly upregulated in paclitaxel-resistant MCF-7/PTX and MDA-MB-231/PTX cell lines [29]. Additionally, FTH1P3 was shown to enhance ABCB1 protein expression by targeting miR-206 [29]. The lncRNA NR_024549 (MRUL), which mediates MDR to doxorubicin and vincristine by upregulating P-gp expression in gastric cancer cells, is also significantly overexpressed in doxorubicin-resistant MCF-7 BrCa cells [30].

In BrCa, altered methylation patterns have been identified in the promoters of *ABCB1*, *ABCC1*, and *ABCG2*, and David *et al*. demonstrated through genome sequencing that the methylation level of the ABCB1 promoter is significantly lower in doxorubicin-resistant MCF-7/ADR cells compared to MCF-7 cells [31, 32]. Furthermore, Dejeux *et al*. investigated methylation patterns in the promoter regions of 14 genes within BrCa tissues. They found that over half of the samples exhibited hypomethylation of *ABCB1*, which was associated with a response to doxorubicin treatment and may contribute to MDR in BrCa [33]. Therefore, it is essential to explore the epigenetic regulation patterns of various ABC transporter proteins in greater depth, as this could enhance our understanding of MDR mechanisms in BrCa and aid in identifying potential therapeutic approaches.

### 3.3 Cell Membrane Lipid Composition and LipidMetabolism

ABC transporter proteins represent a crucial class of transmembrane transporters. Variations in phospholipids and cholesterol, the main components of cell membranes, can significantly affect their transport function (Fig. **2**). Generally, the ABC family of transporter proteins operates through an alternating access mechanism, where the substrate-binding pocket of the transmembrane domain (TMD) undergoes a conformational change, and the substrate is extruded from the cell during the transition from the IF state to the OF state [3]. However, in addition to ATP hydrolysis for energy supply and substrate-binding-induced conformational alterations, it has been observed that peripheral lipid bilayers play a role in the translocation process of these transporter proteins. Moreover, the "annular lipids" shell on the cell membrane is ordered around the protein in a conformation-dependent manner, and its interaction with the transmembrane helical structure of the transporter proteins may affect the alternate entry mechanism [34], suggesting that the function of ABC transporter proteins may be influenced by membrane lipids.

Cholesterol has been proposed to play a regulatory role in the interactions between P-gp and the membrane (Fig. **2**). This regulation facilitates conformational changes within the nucleotide-binding domains (NBDs) of P-gp, enhances the rate of ATP hydrolysis, and ultimately improves the efficiency of P-gp-mediated transport. The lipid environment surrounding the transporter protein is crucial for maintaining its low-energy, stable conformation [35]. Short acyl chain phospholipids destabilize the ABC transporter, resulting in significant ATP hydrolysis. Conversely, long acyl chain phospholipids stabilize the ABC transporter and reduce its ATPase activity [36]. Cholesterol also influences the transport activity of ABC transporters. Following cholesterol intervention in the lipid environment around the ABCC4 transporter, it was observed that cholesterol not only altered the structure of ABCC4 (modified the structure of NBD1) but also regulated its activity [37]. Beyond this, the depletion of cholesterol adversely affects the energy transduction between the NBD and TMD of BCRP [35], and both wild-type BCRP and its R482G mutant require cholesterol for full activity [38]. During lipid metabolism, certain metabolic enzymes are involved in regulating the expression of ABC transporter proteins. One of the key enzymes involved in fatty acid oxidation, long-chain acyl-CoA synthetase 4 (ACSL4), is highly expressed in BrCa cells and regulates the expression of BCRP, through the mTOR pathway in the MCF-7 cell line [39].

### 3.4 EMT

EMT-related markers or transcription factors can regulate the expression and function of ABC transporter proteins (Fig. **2**). For instance, the promoters of several genes encoding ABC transporter proteins contain binding sites for EMT transcription factors, including SNAIL, TWIST, and ZEB. Snail overexpression induces the expression of P-gp and BCRP. Snail possesses the ability to bind to the E-box sequence located on the promoter of E-cadherin through its zinc finger domain. This interaction leads to the suppression of E-cadherin expression and subsequently initiates EMT [5]. Furthermore, a Snail-transfected MCF-7 cell line was found to obtain a high P-gp expression level and MDR after treatment with doxorubicin in BrCa [40].

In addition, ZEB1 facilitates the acquisition of the MDR phenotype while also enhancing their migration and invasion ability. After validation *in vivo* and *in vitro* experiments, ZEB1 was found to upregulate the expression of transporter proteins such as MRP1, BCRP, and ABCC4 in BrCa [5], thereby contributing to the efflux of antitumor drugs and exacerbating MDR in BrCa cells. Conversely, metabolic remodeling-essential for maintaining the EMT phenotype, affects the function of ABC transporter proteins (Fig. **2**) [5, 41]. The progression of EMT in BrCa is accompanied by aerobic glycolysis and overexpression of key glycolytic enzymes. The expression and activation of pyruvate kinase M2 (PKM2), a rate-limiting enzyme in glycolysis, can drive EMT through the PI3K/AKT pathway, where elevated intracellular levels of ATP provide more fuel for ABC transport proteins to excrete drugs [5, 42]. In conclusion, understanding the mechanistic relationship between EMT and MDR phenotypes may offer a foundational basis for developing therapeutic strategies aimed at targeting MDR in BrCa.

### 3.5 Microenvironmental Properties and Composition

#### 3.5.1 Hypoxia

**Fig. (3):**
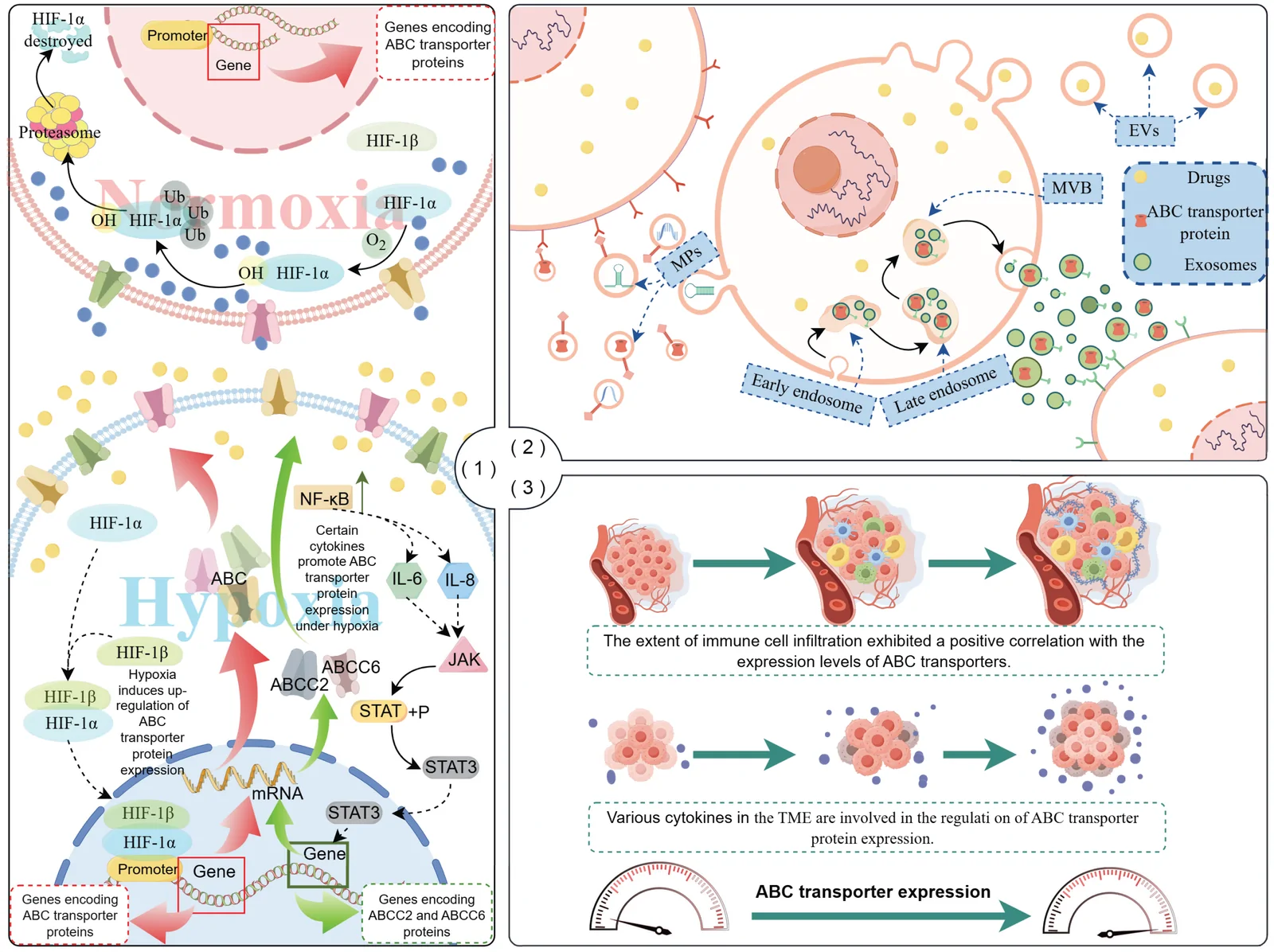
Several factors affect the expression and function of ABC transporter in the tumor microenvironment. (1) Stable HIF-α in a hypoxic microenvironment up-regulates the expression of the ABC transporter, and hypoxia stimulates cytokine production to induce the ABC transporter's expression. (2) Extracellular vesicles excrete drugs, reduce intracellular drug concentration, and act as a transport vehicle to transfer drug-resistant proteins and other substances between cells. (3) Some non-tumor cells and secretory factors in the tumor microenvironment up-regulated the expression of ABC transporter.

HIF-1α plays a pivotal role in the MDR of BrCa by modulating the expression of ABC transporter proteins (Fig. **3**) [43]. Notably, genes encoding specific ABC proteins contain hypoxia-responsive elements within their promoters, which are sensitive to transcriptional regulation by HIF-1α [43]. The hypoxia-responsive element of the gene encoding MRP1 is located 373-378 bases upstream of the ORF, and deletion of this site prevents hypoxia-dependent activation of the *ABCC1* gene [44]. The hypoxia-responsive element of the gene encoding BCRP is located 116 bases upstream of the open reading frame and is induced by hypoxia [44]. Activation of HIF-1α enhances the expression of transporter proteins, thereby increasing drug efflux. For instance, upregulation of ABCB1 expression in response to hypoxia leads to an augmented efflux of doxorubicin from BCSCs [45].

In addition to hypoxia-induced activation of ABC transporter genes, certain cytokines secreted under hypoxia can enhance the expression of ABC transporter proteins. Ossovskaya *et al*. demonstrated that nuclear factor κB (NF-κB) levels were significantly elevated in TNBC compared to normal breast tissue [46]. NF-κB was found to be upregulated under hypoxic conditions and activated through the LPA-LPAR-EZH2-NF-κB signaling pathway, resulting in autocrine secretion of IL-6 and IL-8. The activation of IL-6 and IL-8 ligands leads to JAK transphosphorylation, followed by phosphorylation of STAT monomers; subsequently, activated STAT3 within the nucleus can induce the upregulation of ABCC2 and ABCC6 expression. This process ultimately results in a decrease in intracellular concentrations of antitumor drugs and contributes to chemotherapeutic resistance [28]. Furthermore, Krishnamurthy *et al*. reported that BCRP, a marker for stem and progenitor cells, confers a significant survival advantage in hypoxia, where BCRP expression is upregulated, and that progenitor cells from ABCG2-expressing mice have a stronger survival ability than those from mice that do not express BCRP [47]. Therefore, regulating the hypoxic environment in BrCa and inhibiting the activation of ABC transporter protein-related signaling pathways to reverse MDR in BrCa cells are of exploratory value.

#### 3.5.2 Extracellular Vesicles (EVs)

EVs can induce MDR by inducing the encapsulation of cytotoxic and targeted drugs in BrCa cells and then shedding them outside the cell, leading to a reduction in the intracellular concentration of effective drugs (Fig. **3**) [48]. In addition, EVs contribute to chemoresistance through the delivery of ABC transporter proteins [48]. Specifically, EVs that carry P-gp mediate the transition of BrCa from a drug-sensitive phenotype to a multidrug-resistant phenotype. Lv *et al*. constructed a doxorubicin-resistant MCF-7 cell model and extracted exosomes from its supernatant, which were then co-cultivated with drug-sensitive MCF-7 cells. They found that drug-sensitive MCF-7 cells showed an increase in P-gp expression, which was significantly correlated with the number of exosomes [49]. Additionally, miRNAs loaded within tumor-derived EVs have been shown to regulate the expression of ABC transporter proteins. Jaiswal *et al*. demonstrated that microparticles (MPs) secreted by doxorubicin-resistant MCF-7 cells, which express elevated levels of P-gp, were encapsulated with a significant abundance of transcripts corresponding to the transporter proteins, specifically miR-27a and miR-326. Co-cultivating these MPs with sensitive cells enabled the transfer of transporter protein transcripts, leading to drug-resistant phenotypes in recipient cells, similar to those of the donor cells [50]. Importantly, EVs also interact with various factors that influence ABC transporter proteins; for instance, EMT enhances EV release from cells while simultaneously initiating EMT processes in epithelial cells and promoting EMT development [48]. Therefore, attenuating the delivery of EVs may be a promising strategy for reducing MDR in BrCa cells.

#### 3.5.3 Non-tumor Cells and Secreted Factors in the TME

The impact of non-tumor cells, including macrophages, fibroblasts, and various immune cells, as well as various extracellular components such as growth factors, cytokines, and hormones, on ABC transporter protein-associated BrCa MDR cannot be disregarded (Fig. **3**). Notably, high levels of expression for two ABC transporter proteins-ABCA1 and ABCG1-were detected in cholesterol-loaded macrophages and monocytes [51]. M2 macrophages exhibited an enrichment of liver X receptors that inhibit the SREBP2 signaling pathway when intracellular cholesterol concentrations are elevated. This inhibition subsequently promotes the expression of ABCA1 and ABCG1 transporter proteins [51]. Estimates regarding the abundance of immune cell infiltration conducted by Zhou *et al*. indicated a close association between tumor immune cell infiltration and ABC transporter proteins. Furthermore, the infiltration of B cells, CD8+ T cells, CD4+ T cells, macrophages, neutrophils, and dendritic cells was found to be positively correlated with the expression levels of MRP1, ABCC4, and ABCC5 [52].

Various cytokines within the TME play a crucial role in regulating the expression of ABC transporter proteins. The activation of the epidermal growth factor receptor (EGFR) by epidermal growth factor (EGF) induces the expression of *ABCC1*, *ABCC3*, *ABCC5*, and *ABCC7*-genes that encode ABC transporter proteins, resulting in an increase in ABCC1 promoter activity in MCF-7 cells [53]. Das *et al*. reported that osteopontin (OPN), which is abundantly present in the extracellular matrix, causes intracellular aggregation of glioma-associated oncogene transcription factors through phosphorylation and subsequent inactivation of glycogen synthase kinase-3β (GSK3β). This process initiates the Hedgehog signaling pathway, leading to upregulation of ABCB subfamily transporter protein expression and contributing to resistance against doxorubicin, paclitaxel, and cisplatin in the BrCa MCF-7 cell line [54]. In addition, various other factors are involved in upregulating ABC transporter protein expression in BrCa cells. The combination of interleukin-1β (IL-1β) and estrogenic drugs significantly elevated the mRNA expression levels of ABCC2 and nuclear factor κB (NF-κB; p65) and upregulated the expression of ABCC2 protein in MCF-7 cells [55]. Other soluble factors in TME, such as NF-κB, transforming growth factor β (TGF-β), and insulin-like growth factor II (IGF2), have been found to affect the transcriptional regulation of BCRP in BrCa through multiple signaling pathways [56]. Consequently, upstream factor modulators that regulate the function of MDR-associated non-tumor cells and influence the expression and function of ABC transporter proteins in the BrCa TME are beneficial for overcoming BrCa MDR.

## 4 ABC TRANSPORTER PROTEIN-MEDIATED RE-SENSITIZATION STRATEGIES FOR MDR IN BRCA

Under the influence of various confounding factors, ABC transporter proteins exhibit abnormal expression or possess an active efflux function in BrCa cells. This phenomenon results in an increased intracellular efflux of diverse cytotoxic drugs, enabling BrCa cells to develop drug resistance and progressively evolve into MDR phenotypes, ultimately leading to treatment failure for BrCa. Consequently, targeting ABC transporter proteins and influencing factors is a feasible strategy for reversing MDR in BrCa. This includes targeting of the molecular regulatory network of ABC transporter protein expression, targeting BCSCs, intervention in membrane lipid composition and the BrCa microenvironment, development of novel ABC transporter protein inhibitors, and innovative drug delivery modalities.

### 4.1 Molecular Regulatory Networks Interfering with ABC Transporter Protein Expression

#### 4.1.1 Genetic Modification

Correcting defective genes and regulating gene expression through genetic modifications have emerged as promising therapeutic strategies for reversing MDR. The CRISPR/Cas9 gene editing technology is employed for the knock-in or knock-out of specific genes and has been successfully utilized to address ABC transporter protein-mediated MDR in BrCa. Ha *et al*. utilized the CRISPR/Cas9 system to modify the *ABCB1* gene in MDR BrCa cells and introduced the Cas9-sgRNA system into cells by transfection of a Cas9-sgRNA plasmid, liposome-mediated delivery of RNA-protein (RNP) complexes, and TAT (one of the structural domains of protein transduction)-mediated delivery of complexes. The introduction of the Cas9-sgRNA system into MCF-7/ADR-resistant cells demonstrated an enhancement in doxorubicin efficacy within multidrug-resistant cells, which was attributed to a partial reversal of resistance achieved by disrupting *ABCB1* [57]. Therefore, the knockdown of ABC transporter protein-related genes using CRISPR/Cas9 is a promising approach to overcome MDR in BrCa.

Ribozymes are small RNA molecules with catalytic functions that specifically degrade mRNA sequences by binding to target RNAs and catalyzing RNA splicing reactions. Various ribozymes have been shown to downregulate the expression of ABC transporter protein genes in a sequence-specific manner [58]. Gao *et al*. synthesized two ribozyme genes, RZ106, and RZ135, targeting P-gp mRNA, which were subsequently transfected into doxorubicin-resistant BrCa cells [59]. The results showed that the mRNA and protein expression of P-gp were significantly reduced after transfection with RZ106 and RZ135 [59]. The efflux function of P-gp was inhibited and partially reversed the MDR phenotype of BrCa cells [59]. For multiple ABC transporter proteins, Kowalski *et al*. developed a "multitarget multiribozyme" (MTMR) composed of three trans-acting hammerhead-like nucleases and applied it to a tumor cell model that overexpresses multiple ABC transporter proteins. They observed that the MTMR could specifically down-regulate the expression of the corresponding ABC transporter proteins at both the mRNA and protein levels, effectively reversing drug resistance to selected drugs partially [60]. Controlling the mRNA expression levels of ABC transporter proteins using ribozymes may be a promising strategy for improving MDR in BrCa.

Antisense oligonucleotides (AS-ODNs) are single-stranded DNA oligonucleotides that complementarily bind to gene transcripts, leading to mRNA degradation in the presence of Ribonuclease H1 (RNaseH1), thereby inhibiting protein expression. AS-ODNs have been investigated for the downregulation of *ABCB1* expression, and combination therapy with AS-ODNs and miR-10b could be an important approach toward reversing drug resistance in BrCa [61]. The modification or processing of AS-ODNs has led to the development of various novel oligonucleotide constructs; for instance, hybridization of AS-ODNs with complementary RNA (cRNA) can form heterodimeric double-stranded oligonucleotides with twice the negative charge [62]. From this perspective, the development of AS-ODNs with more potent functionality is valuable for enriching anti-ABC transporter protein-mediated MDR in BrCa as a gene therapy tool.

RNA interference (RNAi) is an important post-transcriptional regulatory mechanism involving the use of small/short interfering RNAs (siRNAs). In BrCa, the application of siRNA to silence P-gp in multidrug-resistant cells has demonstrated a significant enhancement in chemotherapy sensitivity when combined with paclitaxel. Notably, siRNA-mediated cleavage of *ABCB1* gene transcripts simultaneously increased sensitivity to two chemotherapeutic agents, doxorubicin and paclitaxel [58]. In addition to siRNAs, short hairpin RNAs (shRNAs) can also enhance MDR in BrCa by targeting specific gene transcripts and subsequently blocking protein translation [56, 63]. Gan *et al*. downregulated the mRNA level of *ABCB1* using shRNA in the human MCF-7/ADR cell line, which consistently and efficiently reduced the expression of multidrug-resistant genes and restored the sensitivity of drug-resistant cancer cells to conventional chemotherapeutic agents [63]. Gene silencing using siRNAs or shRNAs at the gene expression level of the ABC transporter protein family to modulate the mRNA levels has been reported to be useful against MDR in tumors.

#### 4.1.2 Targeting Upstream Regulatory Factors

Co-expression of multiple ABC transporter proteins may significantly contribute to the refractory nature of MDR in BrCa. Additionally, upstream transcriptional regulators that target ABC transporter proteins could simultaneously influence the expression of several transporter proteins. In TNBC, activated STAT3 has been shown to induce the upregulation of ABCC2 and ABCC6 expression, resulting in chemoresistance at reduced intracellular concentrations of antitumor drugs. Conversely, knockdown of STAT3 resulted in a marked decrease in the expression levels of ABCC2 and ABCC6, which partially reversed cisplatin resistance in TNBC [28]. The eukaryotic initiation factor 4F (eIF4F) complex serves as a key regulator of mRNA translation and regulates the expression of P-gp, MRP1, and BCRP in MCF-7/ADR cells. Pan *et al*. transfected miR-503 mimics into MCF-7/ADR cells, observed significant downregulation of eIF4G and ABC transporter protein expression, and re-sensitized the cells to chemotherapeutic agents such as doxorubicin and tamoxifen [64]. Furthermore, ceramide synthase 4 (CerS4) was found to be overexpressed and activated signaling pathways, including NF-κB, mTOR, and β-catenin, while concurrently upregulating several genes associated with ABC transporters, such as *ABCB1*, *ABCC1*, *ABCC2*, and *ABCC4* [65]. Downregulation of ABC transporter proteins and a reduction in chemotherapeutic resistance were observed after knockdown of CerS4 [65]. CerS4 may be an attractive target for alleviating drug resistance in luminal subtype BrCa.

Similarly, molecules with transporter protein-specific regulatory roles are of interest. For example, Fultang *et al*. reported that receptor tyrosine kinase-like orphan receptor-1 (ROR-1), an upstream regulator of P-gp in BrCa, upregulates P-gp at the transcriptional and protein levels *via* MAPK/ERK and p53, and that ROR1 inhibition results in diminished P-gp transport function and reduced drug efflux [66]. Overall, interference with regulators upstream of ABC transporter proteins has wonderful potential for use in MDR BrCa therapy.

### 4.2 Reversing BrCa MDR by Targeting Variables Affecting ABC Transporter Proteins

During the progression of BrCa, various factors significantly impact the expression and functionality of ABC transporter proteins. These factors include BCSCs, cytosolic lipid composition, and lipid metabolism, all of which contribute to the acquisition of MDR by BrCa cells. Consequently, it is essential to develop targeted therapies that address these influencing factors in order to effectively reverse MDR.

#### 4.2.1 Targeting a Subset of Cells that Aberrantly Express ABC Transporter Proteins - BCSCs

In addition to inhibiting the expression and function of ABC transporter proteins in BCSCs, reducing the population of BCSCs or inducing their differentiation represents an important strategy for overcoming MDR associated with these cells. The ABC transporter proteins, particularly BCRP-an important marker of CSCs-are highly expressed on BCSCs and can be inhibited to reverse drug resistance. Curcumin has been shown to sensitize BCSCs to mitomycin C (MMC) by downregulating the expression of BCRP and MRP1 proteins in MCF-7 and MDA-MB-231 cell lines by factors by 5-fold and 15-fold, respectively [66]. Furthermore, curcumin was also able to reduce the number of BCSCs, in addition to the selective BCRP inhibitor, fumonisin C, which was also found to reduce the survival of BCSCs to a similar extent as curcumin. Bonetti *et al*. demonstrated that miR-34a inhibits the self-renewal of BCSCs and induces their differentiation [67]. After transfection of the triple-negative mesenchymal stromal-like BrCa cell line SUM159PT, which has the characteristics of BCSCs and a low level of miR-34a, with doxycycline-inducible pSlik34a, activation of miR-34a was found to inhibit the proliferation of SUM159PT cells, upregulate the luminal differentiation marker CD24, and downregulate the expression of mesenchymal markers such as N-cadherin and vimentin, suggesting that miR-34a activation induces luminal-like differentiation and suppresses CSC traits [67]. Lv *et al*. identified a negative correlation between the expression levels of glutathione S-transferase Mu (GSTM) and the stemness of BCSCs in TNBC. Furthermore, the upregulation of GSTMs was found to partially diminish BCSC enrichment [68]. In addition, salinomycin-a potassium ion carrier- has been identified as a selective inhibitor of human BCSCs, targeting the inhibition of BCSC proliferation and induction of differentiation [69].

Several studies have shown that BCSC self-renewal signaling pathways can be targets for depleting the BCSC population, and targeting these pathways may be a rational approach toward improving MDR in BrCa. The Notch signaling pathway is crucial for maintaining the stem cell characteristics of BCSCs, and evidence suggests that the Notch inhibitor DAPT can reduce the stem cell phenotype of the brain metastatic BrCa cell line MDA-MB-231 (231-BR) *in vitro*, thereby exerting antiproliferative effects [70]. BrCa cells with stem cell properties exhibit high STAT3 activity. Ko *et al*. found that tangeretin disrupted the STAT3/SOX2 signaling pathway, which indirectly downregulated the RNA and protein levels of SOX2 by decreasing the total protein levels of STAT3 and pSTAT3 in BCSCs, ultimately inhibiting the proliferation of BCSCs [71]. Lv *et al*. employed digoxin to target HIF-1/CBS, thereby inducing ferroptosis to decrease the population of BCSCs in TNBC [72]. In addition to the above pathways, signaling pathways such as PI3K/

AKT, Wnt/β-catenin, and Hippo/YAP can promote BCSC self-renewal, stemness, and drug resistance, and can be regarded as therapeutic targets for the development of therapies to modulate BCSCs, and ultimately reduce ABC transporter protein-mediated drug efflux to reverse MDR.

#### 4.2.2 Intervening Membrane Lipid Composition and the BrCa Microenvironment

Modulation of membrane lipids surrounding ABC transporter proteins represents a promising strategy for inhibiting protein function and reducing drug efflux, thereby reversing MDR in BrCa cells. Certain ABC transporter proteins that co-localized with cholesterol-rich microstructural domains are found within lipid rafts; thus, the regulation of these lipid rafts can indirectly affect the activity of ABC transporter proteins. Azurin, a member of the redox protein family, is a copper-containing periplasmic protein secreted by *Pseudomonas aeruginosa* that has been investigated as a drug candidate in BrCa therapy. A close interaction between Azurin and membrane lipid rafts alters the expression levels of caveolar proteins at the plasma membrane and affects lipid raft exposure [73]. In addition, modifications in the composition of membrane phospholipids can impact the microstructural domains of membranes surrounding ABC transporter proteins, potentially influencing the activity of these transporters [74]. Xue *et al*. reported that polyunsaturated fatty acids increase oxidative stress and enhance the sensitivity of BrCa cells to doxorubicin [75].

In addition, several HIF-1 inhibitors including acriflavine, oroxylin A, and echinomycin, have the potential to be developed for the inhibition of ABC transporter protein expression by ameliorating the hypoxic microenvironment [76, 77]. In both MDA-MB-231 and MDA-MB-435 cell lines, acriflavine effectively inhibited HIF-1 activity at the transcriptional level by preventing the dimerization of HIF-1α and HIF-1β [76]. The natural product oroxylin A, a flavonoid derived from *Scutellaria baicalensis*, induced HIF-1α degradation through the prolyl hydroxylase-VHL signaling pathway [77]. Another natural product, Baicarein, inhibits HIF-1α to reverse mitochondrial dysfunction and re-sensitize tamoxifen-resistant BrCa cells [78]. Furthermore, the antibiotic drug echinocandin, a small-molecule inhibitor of HIF-1, inhibits the function of HIF-1 by reducing the binding of HIF-1α to DNA. In addition to the inhibitors mentioned above, many small-molecule inhibitors have been developed based on HIF-1 under investigation, such as chetomin, bortezomib, and the compound LW1564 [43, 79]. The complexity of the TME necessitates more comprehensive intervention strategies. The emergence of biological therapy offers a novel perspective for TME modulation. Cai *et al*. demonstrated that cyanobacteria, naturally occurring organisms, produce oxygen *via* photosynthesis under laser irradiation, thereby altering the hypoxic microenvironment in BrCa. Following the alleviation of hypoxia, the expression of hypoxia-inducible factor was downregulated, which in turn inhibited the expression of P-gp and reduced the efflux of doxorubicin in BrCa cells [80].

### 4.3 Drug Development Targeting ABC Transporter Proteins

#### 4.3.1 "New Use of Old Medicine" - Drug Reuse

Exploring novel applications for "old drugs" is crucial for the more efficient development of ABC transporter protein inhibitors. This approach aims to mitigate the high cost and risks associated with new drug development while expanding the therapeutic indications of existing medications. Researchers are actively investigating alternative uses for established drugs beyond their indications to enhance MDR by targeting ABC transporter proteins (Table **1**). Sulbactam, an irreversible competitive inhibitor of β-lactamase, is utilized in the treatment of bacterial infections. It has been shown to reduce the expression levels of ABCB1, ABCB5, and ABCG2 in MDA-MB-453 and MDA-MB-468 cell lines. This inhibition effectively suppresses the efflux of doxorubicin, thereby prolonging its retention time within BrCa cells [81]. Furthermore, Park *et al*. discovered that pimecrolimus, a commonly prescribed medication for inflammatory skin diseases and a calmodulin-dependent phosphatase inhibitor, inhibits the efflux of vincristine (VIC) from multidrug-resistant MCF-7/ADR cells by directly binding to P-gp transporter proteins. This interaction enhances the cytotoxic effects of VIC in MDR cells [82]. Additionally, vitamin C, a widely recognized antioxidant has been found to reduce drug efflux from doxorubicin-resistant MCF-7/DOX BrCa cells by inhibiting P-gp expression and decreasing ATP levels [83]. Consequently, this resensitizes resistant cells to doxorubicin treatment.

In addition to expanding the range of indications for approved drugs, structural modification of antitumor drugs or ABC transporter protein inhibitors to avoid extrusion of the drug out of the cell or enhancement of the inhibitor's effect on ABC transporter protein expression or transport is a way of circumventing or reversing MDR in BrCa cells. In a previous study, researchers modified a phenylthiophene (pyridine) derivative with P-gp inhibitory effects by introducing methoxy groups into the benzene ring; multiple substitutions of the benzene ring enhanced the inhibitory effects of the pyridine derivative on P-gp [84]. The modified compound was tested in multidrug-resistant MCF-7/ADR cells, resulting in a substantial increase in intracellular concentrations of Rhodamine123 (Rh123). Further investigations demonstrated that this compound could serve as a chemical sensitizer for cytotoxic drugs such as doxorubicin, paclitaxel, vincristine, and zorubicin within MCF-7/ADR cells [84, 85]. Similarly, compound 12c, with a Y-shaped structure synthesized by Zhang *et al*. using Naamidines as a structural scaffold, effectively reversed paclitaxel resistance in the multidrug-resistant cell line MDA435/LCC6MDR [86]. To enhance the tissue specificity of P-gp inhibitors in MDR BrCa, Li *et al*. proposed a novel folic acid compound that combines the targeting of folic acid to BrCa with the MDR reversal activity of a novel phenylfuran P-gp inhibitor. Subsequent experiments demonstrated that when this compound was co-administered with doxorubicin hydrochloride in MCF-7/ADR cell lines, it significantly potentiated the efficacy of doxorubicin hydrochloride [87]. Drug modifications have been directed towards antitumor drugs in addition to ABC transporter protein inhibitors, the development of drugs with non-transporter protein substrate characteristics, or dual-acting drugs for circumventing ABC transporter protein-mediated MDR [85]. This approach has been practiced on human leukemia cells (HL-60), where Długosz-Pokorska *et al*. designed methylenedihydrouracil analogs of 5-fluorouracil (5-FU) and used them against a drug-resistant cell line (HL-60/5FU) [88]. In contrast with the 5-FU, analogous compound could not be pumped out of the cell *via* the ABC transporter protein, but could inhibit the function of the transporter proteins, making this approach worth trying in multidrug-resistant BrCa [88].

#### 4.3.2 Fourth Generation Inhibitors - Natural Products

The development of inhibitors targeting ABC transporter proteins has undergone several iterations, with researchers exploring the potential of approved drugs as inhibitors for these transport proteins. Notable examples include verapamil, cyclosporine A, trifluoperazine, quinidine, and luteinizing hormone [89]. However, none of these agents have been successfully implemented in clinical settings due to their poor specificity, low affinity, toxicity, and high incidence of adverse effects [89]. Despite the unsatisfactory outcomes from initial investigations, efforts to develop ABC transporter protein inhibitors remain active. Researchers are now focusing on natural products that offer advantages such as multi-targeting capabilities and reduced toxicity. These compounds are being explored for the advancement of fourth-generation ABC transporter protein inhibitors. Various compounds, including flavonoids and alkaloids, have been found to have an inhibitory effect on the function of ABC transporter proteins in MDR BrCa cells (Table **1**).

Flavonoids have the capacity to bind to the NBD of ABC transporter proteins and interact with the TMD, leading to alterations in protein conformation that ultimately reduce drug efflux. Various flavonoids, such as kaempferol, quercetin, and apigenin, have been found to modulate transporter proteins, such as P-gp, MRP1, and BCRP. Chung *et al*. found that MCF-7/ADR cells overexpressing the P-gp, kaempferol, and its derivatives increased the intracellular accumulation of [3 H]-daunomycin (DNM) by inhibiting P-gp transporter protein-mediated drug efflux; in particular, the introduction of hydrophobic motifs at the 3-position of the C-ring may be important for the inhibition of P-gp activity [90]. Furthermore, hesperidin was found to decrease both expression levels and activity of P-gp in doxorubicin-resistant BrCa cells, thereby increasing cellular sensitivity to doxorubicin [91]. Additionally, apigenin has been reported to have an inhibitory effect on the colonization and growth of doxorubicin-resistant MCF-7 cells through the inhibition of P-gp and MRP1 function and targeting STAT3 signaling and its nuclear translocation [92].

Several studies have shown that alkaloids serve as substrates for P-gp, MRP1, and BCRP. Some of these alkaloids exhibit inhibitory effects on P-gp activity due to the structural characteristics of their basic nitrogen atoms and planar aromatic rings; consequently, they can function as competitive inhibitors to mitigate drug efflux [93]. More lipophilic isoquinolines, indoles, and steroidal alkaloids have stronger binding capacities to ABC transporter proteins than the more polar pyridines and scopoletanes. Berberine, which belongs to the isoquinoline alkaloids, inhibits the P-gp function and enhances the sensitivity of doxorubicin-resistant BrCa cells by downregulating the AMPK-HIF-1α signaling pathway [94]. In addition, the amide alkaloid piperine downregulates P-gp, MRP1, and BCRP expression and differentially reverses resistance to doxorubicin and mitoxantrone in the multidrug-resistant cell line MCF7/DOX [95]. In addition to the flavonoids and alkaloids mentioned above, various natural products, including coumarins, terpenoids, and lignans, which have significant potential for the development of inhibitors, can function as ABC transporter protein inhibitors to reverse MDR in BrCa cells.

Natural products derived from plants have demonstrated tremendous potential in the development of drugs targeting ABC transporters to reverse MDR in BrCa. Nevertheless, it is notable that there are also abundant compounds in the metabolites of microorganisms. Thus, compounds of microbial origin constitute an important resource pool for drug development. Qian *et al*. carried out the fermentation culture of Tubeufia rubra PF02-2, and extracted, separated and purified the fermentation products. Eventually, the glyceroglycolipid compound PF26 was obtained [96]. On this basis, the researchers employed the glyceroglycolipid compound PF26 to prepare a transport pump inhibitor and applied the inhibitor to MCF-7/ADR cells. It was discovered that this inhibitor exerted an inhibitory effect on one or several of the ABC transporters [96]. In a nutshell, compounds of microbial origin provide an alternative perspective for the development of ABC transporter inhibitors.

#### 4.3.3 Optimizing Drug Delivery Systems

Traditional drug delivery mainly relies on the non-targeted transport of small-molecule drugs within biological fluids. This approach often encounters significant drawbacks, including high drug toxicity to non-targeted tissues, and limitations in efficacy due to challenges in controlling the solubility and distribution of the drugs. In the case of highly heterogeneous BrCa, overcoming the development of drug resistance through conventional drug delivery methods proves to be particularly challenging. However, the emergence of nanodrug delivery systems presents new opportunities for reversing MDR in BrCa.

Nanoparticle-based drug delivery has the advantages of improving drug stability and targeting, reducing drug side effects, and decreasing drug clearance. Numerous nanocarriers have been reported for their role in drug resistance, such as nanocarriers combining MDR-reversing and cytotoxic drugs, targeted nanoparticles to improve drug specificity, and nanocarriers that circumvent or inhibit ABC transporter proteins to increase intracellular drug accumulation. Narayanan *et al*. prepared PLGA-casein core/shell nanoparticles, where paclitaxel was encapsulated with the flavonoid epigallocatechin gallate, and used them for the treatment of paclitaxel-resistant MDA-MB-231 cells, showing that the combination downregulated the expression of P-gp at both the gene and protein levels and re-sensitize the resistant cells to the drug [97]. Similarly, Liu *et al*. fabricated HSA-MnO2 nanoparticles with human serum albumin (HSA) serving as a template scaffold and loaded the drug doxorubicin, obtaining DOX/HSA-MnO2 nanoparticles. The DOX/HSA-MnO2 nanoparticles exhibit photothermal stability and oxygen-producing traits. Consequently, when combined with photothermal therapy, they can markedly reduce the expression levels of HIF-1α and P-gp in cells, effectively reversing MDR in BrCa [98]. In addition, the siRNAs mentioned in the strategy to regulate the expression of ABC transporter protein genes are limited by poor stability and a high clearance rate during use. One study reported that mixed dendrimer micelle nanocarriers are able to deliver siMDR-1 and doxorubicin to doxorubicin-resistant BrCa cells, and efficiently overcome MDR [99]. In addition to these nanocarriers, many different types of nanocarriers are under development, and the use of nanocarriers to improve ABC transporter protein-mediated MDR in BrCa cells is desirable.

#### 4.3.4 Exploiting Collateral Sensitization from ABC Transporter Protein Overexpression

Certain studies have indicated that the overexpression of ABC transporter proteins may enhance the sensitivity of drug-resistant cells to various pharmacological agents [100, 101]. The MRP1 transporter protein, which functions as a GSH transporter pump, exhibits a negative correlation with intracellular GSH levels and primarily facilitates the efflux of GSH from the cell. This process leads to a decrease in intracellular GSH accumulation-a phenomenon wherein resistance to one class of drugs in tumor cells is associated with increased sensitivity to other classes of drugs [102]. In MDR BrCa cells, MRP1 is highly expressed, and GSH is extruded outside the cell, which stimulates the intracellular synthesis of GSH to maintain a relatively steady state [103]. After adding inhibitors of the GSH synthesis rate-limiting enzyme γ-glutamylcysteine ligase (γ-GCL) to buthionine sulfoximine (BSO) on various tumor cells, researchers found that MRP1-overexpressing cells were more sensitive to BSO than MRP1-under-expressing cells [101]. Targeting the relevant components of the GSH synthesis pathway could provide a therapeutic modality for BrCa cells with an MDR phenotype. Lien *et al*. inhibited the biosynthesis of GSH using BSO, which selectively sensitized BrCa cells with abnormalities in the PI3K/Akt pathway to cisplatin [104].

### 4.4 Joint Application of Multiple Methods

Given the complexity of the mechanism underlying MDR in BrCa, combination therapy is more likely to achieve the desired therapeutic effect compared to monotherapy. Several studies have demonstrated that simultaneous targeting of multiple signaling pathways can enhance the reversal of MDR in BrCa. For example, cyclosporine A, a multi-specific ABC transporter protein inhibitor, has the ability to regulate signaling pathways such as TGF-β and ERK, and is able to inhibit the expression of P-gp, MRP1, and BCRP simultaneously, thereby reducing drug efflux.

The combination of various treatment modalities significantly enhances the capacity of BrCa cells to reverse MDR. Lin *et al*. found that a combination of the natural product baicalin and chemotherapeutic drug doxorubicin improved the chemosensitivity of MDA-MB-231 and MCF-7 cells [105]. In addition, the application of nanocarriers can synergistically enhance the reversal of MDR in BrCa cells. Zhang *et al*. reported a pH- and redox-responsive dual-responsive nanocarrier system for combining the chemotherapeutic drug doxorubicin and cyclooxygenase 2 (COX-2) inhibitor celecoxib for the treatment of MDR in BrCa, which, *in vitro*, enhanced the inhibitory effect of doxorubicin on multidrug-resistant BrCa cells *via* the CD44/HA-specific binding-mediated endocytosis [106]. COX-2 inhibition-induced downregulation of P-gp expression enhanced the inhibitory effect of doxorubicin on MDR BrCa cells, and in the *in vivo* experiments, the nanocarrier with good tumor-targeting properties, reduced the expression of COX-2 and P-gp in tumor tissues, and improved the sensitivity of chemotherapy [106]. In conclusion, the strategy of combination therapy to improve drug resistance in BrCa is worth exploring in depth.

## CONCLUSION AND CURRENT AND FUTURE DEVELOPMENTS

MDR is a prevalent and persistent obstacle in the treatment process of BrCa. Among the various mechanisms that contribute to MDR in BrCa, drug efflux mediated by the overexpression of ABC transporter proteins is the most prevalent. These transporter proteins, located on the cell membrane, bind to therapeutic agents and undergo conformational changes that facilitate the translocation of intracellular antitumor drugs into the extracellular space. This process leads to a reduction in intracellular concentrations of effective medications, thereby contributing to resistance against endocrine therapies and chemotherapeutics. The aberrant expression of ABC transporter proteins associated with MDR acquisition in BrCa cells has been linked to multiple factors, including BCSCs, epigenetic modifications, and membrane lipid composition. Additionally, metabolic characteristics and microenvironmental components have been identified as contributors to the overexpression of ABC transporter proteins at both transcriptional and protein levels. To resolve MDR, which complicates BrCa treatment, finding ways to target ABC transporter proteins is worth considering, including intervention in the molecular networks related to ABC transporter protein expression, and developing drugs and therapeutic approaches targeting these proteins. In addition to directly targeting and regulating the expression and function of ABC transporter proteins, approaches that intervene in ABC transporter-related factors can also potentially inhibit MDR in BrCa.

Various therapeutic strategies have been developed to reverse MDR in BrCa by inhibiting the drug efflux mediated by ABC transporter proteins. These include gene editing technologies such as CRISPR/Cas9, plasmids, and ribozymes to regulate gene expression, drug modification, and the development of novel inhibitors based on natural products, the development of nanomedicine delivery systems, and the design of combination drug regimens. Although these treatments above have effectively targeted ABC transporter proteins to reverse MDR in BrCa cells, many questions remain unanswered owing to the heterogeneity of BrCa and complexity of its resistance mechanisms. For example, when employing ABC transporter inhibitors, it is crucial to establish methods for optimizing the screening process for potential beneficiaries, balancing effective therapeutic doses with minimal toxic side effects, enhancing tissue specificity of the inhibitors, and preventing the emergence of resistance against ABC transporter inhibition. Looking ahead, we need to focus on mining biomarkers related to MDR in BrCa cells, development of more low-toxicity and high-efficiency drugs from natural compounds, application of new therapeutic tools such as immunotherapy and gas molecular therapy, development of a more efficient drug delivery system, and development of clinical trials of gene therapy for the reversal of MDR in BrCa.

## Figures and Tables

**Table 1:** Progress in the study of drug reuse and natural products of ABC transporter inhibitors.

**Group**	**Name**	Chemical Structure^*^	**Mode of Action**	**ABC** **Transporters**	**References**
Drug reuse	Sulbactam	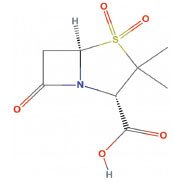	Compete with ATP for the ATP-docking sites of ABCB1, ABCB10, ABCC1	ABCB1, ABCB10, ABCC1	[81]
	Pimecrolimus	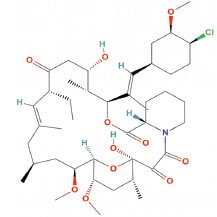	Bind directly to the ABCB1 transporter	ABCB1	[82]
	Pimozide	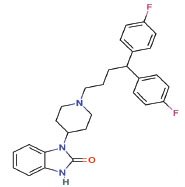	Targeting Signal transducer and activator of transcription 5a, indirectly regulating ABCB1 transporter expression	ABCB1	[107]
	Metformin	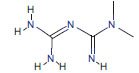	Reduce ABCB1 activity	ABCB1	[108]
	Vitamin C	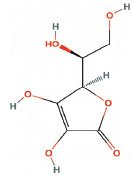	Inhibition of ABCB1 expression and a decrease in ATP levels	ABCB1	[83]
	Vitamin E succinate	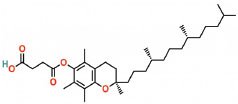	Inhibit the activity of ATPase	ABCB1	[109]
	A phenylthiophene (pyridine) derivative	(No details)	The derivative modification introduced a methoxy group into the benzene ring, while multiple substitutions on the benzene ring enhanced the inhibitory effect of pyridine derivatives on ABCB1.	ABCB1	[84]
	Compound 12c	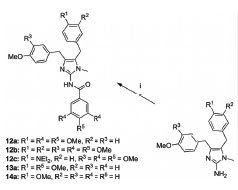	Modulation of ABCB1 to Decrease Drug Efflux	ABCB1	[86]
Natural products	Kaempferol	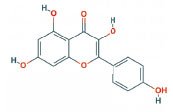	Inhibition of ABCB1 activity	ABCB1	[90]
	Hesperidin	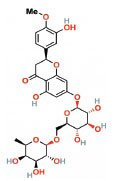	Decreased the expression and inhibited the activity of the P-gp	ABCB1	[91]
	Apigenin	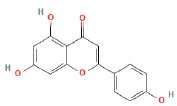	Inhibition of the functions of ABCB1 and ABCC1 transporters	ABCB1, ABCC1	[92]
	Piperine	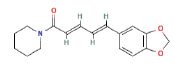	Downregulates ABCB1, ABCC1, and ABCG2 expression	ABCB1, ABCC1, ABCG2	[95]
	Quercetin	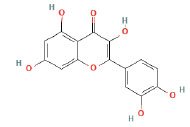	Directly interacts with transporters to inhibit drug efflux mediated by ABCB1, ABRB2 or ABCG2.	ABCB1, ABRB2, ABCG2.	[110]
	Glaucine	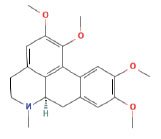	Inhibits ABCB1 and ABCC1-mediated efflux and down-regulates transporter gene expression.	ABCB1, ABCC1	[111]

## LIST OF ABBREVIATIONS

5-FU: 5-Fluorouracil
ABC: ATP-Binding Cassette
ACSL4: Acyl-CoA Synthetase 4
AS-ODNs: Antisense Oligonucleotides
BCRP: Breast Cancer Resistance Protein
BCSCs: Breast Cancer Stem Cells
BrCa: Breast Cancer
BSO: Buthionine Sulfoximine
CerS4: Ceramide Synthase 4
COX-2: Cyclooxygenase 2
cRNA: Complementary RNA
CSCs: Cancer Stem Cells
DNM: Daunomycin
EGF: Epidermal Growth Factor
EGFR: Epidermal Growth Factor Receptor
eIF4F: Eukaryotic Initiation Factor 4F
EMT: Epithelial-Mesenchymal Transition
EVs: Extracellular Vesicles
FTH1P3: Ferritin Heavy Chain 1 Pseudogene 3
GSH: Glutathione
GSK3β: Glycogen Synthase Kinase-3β
GSTM: Glutathione S-Transferase Mu
HIF-1: Hypoxia-Inducible Factor 1
HSA: Human Serum Albumin
ICIs: Immune Checkpoint Inhibitors
IGF2: Insulin-Like Growth Factor II
IL-1β: Interleukin-1β
lncRNAs: Long-Chain Non-Coding RNAs
MDR: Drug Resistance
MMC: Mitomycin C
MPs: Microparticles
MRP1: Multidrug Resistance Protein 1
MTMR: Multitarget Multiribozyme
NAC: Neoadjuvant Chemotherapy
NBDs: Nucleotide-Binding Domains
ncRNA: Aberrant Non-Coding RNA
NF-κB: Nuclear factor κB
OPN: Osteopontin
ORFs: Open Reading Frames
P-gp: P-Glycoprotein
PCT: Polychemotherapy
Rh123: Rhodamine123
RNAi: RNA Interference
RNaseH1: Ribonuclease H1
RNP: RNA-Protein
ROR-1: Receptor Tyrosine Kinase-Like Orphan Receptor-1
SERM: Selective Estrogen Receptor Modulator
SGLT2: Sodium-Glucose Transport Protein 2
shRNAs: Short Hairpin RNAs
siRNAs: Small/Short Interfering RNAs
SP: Side Population
TGF-β: Transforming Growth Factor β
TMD: Transmembrane Domain
TNBC: Triple-Egative Breast Cancer
VIC: Vincristine
γ-GCL: γ-Glutamylcysteine Ligase
